# Optimization of cell culture and cell disruption processes to enhance the production of thermophilic cellulase FnCel5A in *E*.*coli* using response surface methodology

**DOI:** 10.1371/journal.pone.0210595

**Published:** 2019-01-17

**Authors:** Shah Faisal Mohammad, Yan Feng, Guangyu Yang

**Affiliations:** State Key Laboratory of Microbial Metabolism, School of Life Sciences and Biotechnology, Shanghai Jiao Tong University, Shanghai, China; University of Huddersfield, UNITED KINGDOM

## Abstract

FnCel5A from *Fervidobacterium nodosum* is one of the most thermostable endoglucanases that have phenomenal characteristics, such as high activity, pH stability, and multi-specificity towards various substrates. However, large-scale thermophilic enzyme production is still a challenge. Herein, we focus on an optimization approach based on response surface methodology to improve the production of this enzyme. First, a Box-Behnken design was used to examine physiochemical parameters such as induction temperatures, isopropylβ-D-1-thiogalactopyranoside concentrations and induction times on the heterogeneous expression of FnCel5A gene in *E*. *coli*. The best culture was collected after adding 0.56 mM IPTG and incubating it for 29.5 h at 24°C. The highest enzymatic activity observed was 3.31 IU/mL. Second, an economical "thermolysis" cell lysis method for the liberation of the enzymes was also optimized using Box-Behnken design. The optimal levels of the variables were temperature 77°C, pH 7.71, and incubation time of 20 min, which gave about 74.3% higher activity than the well-established bead-milling cell disruption method. The maximum productivity of FnCel5A achieved (5772 IU/L) illustrated that its production increased significantly after combining both optimal models. This strategy can be scaled-up readily for overproduction of FnCel5A from recombinant *E*.*coli* to facilitate its usage in biomass energy production.

## Introduction

The enzymatic conversion of plant biomass into fuels has sparked a keen interest in their potential for solving problems related to climate change, energy security, and global economic development. However, the development of fossil fuel to a biomass-based economy is not easily possible for a number of reasons [1]. The production of biochemical and bioethanol products poses technical and financial problems due to the complex nature of lignocellulose. Improvements in treatment efficiency and financing are required to make these alternative fuels economically viable [2].

Heat-stable lignocellulose degrading enzymes have the potential to overcome these limitations by chemically expediting the production of biofuels [3, 4]. Thermostable cellulases serve as ideal catalysts for this application because high temperatures generally favor more efficient enzymatic digestion of raw materials [5]. In addition, high-temperature enzymatic hydrolysis is more suitable for the commonly used high-temperature pretreatment processes, thus reducing the need for expensive biomass cooling [6, 7]. The use of thermophilic enzymes facilitates various aspects of current lignocellulose to bioethanol process configurations.

Endoglucanases, one of the main cellulase enzymes utilized in biomass processing, randomly hydrolyzes accessible internal β-1, 4-glucosidic bonds in cellulose chains [8]. A thermostable endoglucanase of the GH5 family called FnCel5A catalyzes the hydrolysis of cellulose to glucose in the thermophilic bacterium *Fervidobacterium nodosum* Rt17-B1. It is the first cellulase of the genus *Fervidobacterium* that has been cloned and expressed [9]. FnCel5A is particularly suitable because of its high thermostability (half-life of 48 hours, Topt = 353 K) and its high specificity for carboxymethyl cellulose [10]. It also has a wide range of affinity for the other substrates like β-1, 4-linked polysaccharides, including xyloglucan barley, glucomannan, β-glucan, lichenin, and galactomannan [9]. This combination of thermostability and activity makes FnCel5A particularly suitable for industrial hydrolysis of cellulose, which involves prolonged treatment at high temperatures, as required when converting biomass into biofuels. The low yield and high cost of this enzyme are the major bottlenecks of its industrial applications. To date, little work has been directed to improve this fermentation process [11, 12]. Optimizing the cellulase production approach can decrease the amount of enzyme production to increase the efficiency of biomass processing and thus reduce the cost of cellulosic bioethanol production [13].

Complete cell disruption is crucial to achieve maximum release of intracellular proteins and ultimately facilitates recovery of the protein of interest and subsequent purification [14–16]. A variety of cell disruption techniques have been studied and examined in detail in the literature [17–22]. To be commercially feasible on an industrial scale, a multitude of procedural factors must be considered and optimized, including disruption efficiency, duration, power requirement, recovery efficiency, and productivity [23, 24]. The choice of the disruption method depends on specific treatment parameters, for example the nature of the product released, thermostability, activity, half-life, the tolerance of a range of pH, ionic concentrations and the application considered. Current methods are plagued by low yields, unwanted chemical dependence, and contamination by cell debris, resulting in an inefficient, time-consuming and costly downstream separation process [25, 26]. An optimized recovery protocol should result in a concentrated enzyme extract with minimal loss and downstream processing, which would increase commercial viability [27–29].

Studies that report high yields in enzyme production and bioprocessing have used response surface methodology (RSM) to optimize their experimental parameters [30, 31]. RSM is a collection of statistical and mathematical applications that model the effects of individual factors and their interactions. This methodology is employed to solve multivariable equations and to simultaneously evaluate the relative importance of several input variables in complex systems towards a desired outcome. This is achieved by multiple regression analysis using quantitative data collected from appropriately designed tests. [18]. Based on the preferred features for orthogonality and rotation ability central composite design (CCD) and Box-Behnken design (BBD) are generally used for model optimization [19]. BBD is the most frequently applicable three-level fractional factorial design in the creation of second-order response surface models.

The present study aimed to optimize the culture and disruption techniques integral to FnCel5A production. Highly efficient and effective extraction of this endoglucanase was achieved through modulation of physical and chemical parameters using RSM based on the BBD. This study provides appropriate and optimized modules for the efficient production of thermophilic cellulases, which will contribute to the production of biofuels at the industrial level. In addition, this process is more facile, cost-effective, and less time consuming and as such, it will result in an improvement to the overall economy.

## Materials and methods

### Materials and strain

All chemicals were purchased from (Oxoid Ltd England or Shanghai Lingfeng Oxoid Ltd chemical Reagent Company Ltd China) unless otherwise stated. Low viscosity carboxymethyl cellulose (CMC) was obtained from Sigma-Aldrich, U.S.A. *E*. *coli* (BL21) for gene cloning and recombinant enzyme production was routinely cultured at 37°C in Luria-Bertani (LB) medium.

### Standard culture conditions

*E*. *coli* (BL21) cells that contain plasmids carrying the pET-15b gene, encoding for recombinant FnCel5A under control of the T7 promoter, were cultured with ampicillin (100 μg/mL) using the shake flask method. For FnCel5A production, cells were cultured in 250 mL shake flasks with 100 mL of LB medium and rigorous shaking (250 rpm) for three hours at 37°C. Gene expression was induced with isopropyl-thio-β-D-galactosidase (IPTG) according to the BBD.1mL cell culture from each flask were then centrifuged at 8,000 ×g for eight minutes at 4°C. Prior to cell disruption, the cell pellet were washed three times with double distilled water and centrifuged at 8000 x g for two minutes at 4°C.

### Glass bead disruption

The washed cells were re-suspended in 1mL of cell breaking buffer (Tris–HCl, pH 8.0). Cells expressing recombinant FnCel5A were then subjected to further cellular disruption with glass beads. The bead milling method [32] was conducted using the High Throughput Tissuelyser (Ningbo Scientz, Biotechnology Co., Ltd, P.R. China) up to eight times (working time 90 seconds, resting time 30 seconds) at room temperature. Each cell sample, suspended in 1 mL of cell breaking buffer, was mixed with 0.5 g of alcohol-washed glass beads in 1.5 mL centrifuge tubes. These conditions were utilized in all experiments, unless otherwise noted. The samples were subsequently analyzed at predetermined time intervals for data collection.

### Total cellulase assay

Total cellulase activity was measured using the 3, 5-Dinitrosalicylic Acid (DNS) method [33]. The hydrolytic activity of the FnCel5A was measured after five minutes of incubation at 80°C in 50 mM phosphate-citrate buffer (pH 5.5) with polysaccharides (1% w/v), specifically CMC (carboxymethyl cellulose sodium salt), low viscosity; prepared by a previous reported method[34]. After incubation, 600μL DNS was added and kept in a 100°C boiling water bath for five minutes. The resulting reduced sugars were measured at 540 nm in an UV–Vis spectrophotometer (Shimadzu) using glucose as a standard. One international unit (IU) of cellulase activity was defined as the amount of enzyme required to release 1 μmol of glucose per minute under standard assay conditions [35].

### Statistical optimization of culture conditions for FnCel5A production

To determine the optimal culture conditions for FnCel5A production, RSM was used with BBD. The culture parameters included in this study are listed in Table 1 with three coded levels (-1, 0, +1). The response value (Y) in each experimental test represents the average of the triplicates. The Design Expert software (version 10.0.6.0, purchased from Stat-Ease USA, Shanghai Branch) was used to design matrices and statistical analysis of the data. Regression analysis was performed to evaluate the response function as a second-order polynomial [36].

**Table 1:** Variable levels for *E*.*coli* culture conditions.

Independent		Variable levels	
	-1	0	+1
A) IPTG (mM)	0.02	0.51	1
B) Temp (°C)	16	23	30
C) Time (h)	10	23	36

Maximum and minimum levels for the variables used in the Box-Behnken design

The validity of the model was verified by analysis of variance (ANOVA) using Design Expert software. The significance of the model was confirmed by the *F*-test (Fisher test). The adequacy and quality of the quadratic equation were statistically verified by the coefficient of determination (R^2^) and the adjusted (R^2^). The three-dimensional response surface plots and their respective contour curves were designed to better understand the correlation between the responses and the experimental levels of each independent variable.

### *E*. *coli* cell disruption

1 g of *E*. *coli* cell paste was resuspended in 10 ml of sodium phosphate buffer (20 mM) in 50 ml Falcon tubes and centrifuged at 8000 xg for 30 minutes to separate the FnCel5A enzyme fraction from the cellular debris. Total enzymatic activity was measured using 3, 5-dinitrosalicylic acid (DNS) as a sugar reducing reagent as described in the previous section. The variable parameters for *E*. *coli* cell disruption including temperature, pH, and incubation time were optimized by RSM with BBD. The levels for the cell disruption parameters investigated in this study are given in Table 2.

**Table 2:** Variable levels for *E*. *coli* cell disruption.

Independent		Variable levels	
	-1	0	+1
A) Temperature(°C)	70	83	96
B) pH	6.2	7.2	8.2
C) Time (minutes)	5	17.5	30

Maximum and minimum levels for the variables used in the Box-Behnken design

## Results and discussion

### Box-Behnken analysis of *E*.*coli* culture

To optimize the *E*.*coli* culturing condition for FnCel5A production, a three-factor, three-level BBD based on RSM was chosen to analyze the effects of all the three parameters: IPTG concentration (A), induction temperature (B), and induction time (C), results were shown in Table 3. Various parameters can influence the production of recombinant proteins, e.g. induction time, IPTG concentration, and post-induction temperature [37]. Box-Behnken's design data for the optimization of the *E*.*coli* was subjected to a second-order polynomial regression analysis using the least squares regression method to get the estimated parameters of the mathematical model. The design include seventeen experimental runs (12 factorial and 5 central) carried out in a random order. Three replicates (runs 2, 13, 14, 1, and 7) at the center of the experimental design were used to elucidate the pure error sum of squares.

**Table 3:** Box-Behnken design arrangement for culture conditions and resulting activity.

Factor 1			Factor 2	Factor 3	Response
Std	Run	A:IPTG (mM)	B:Temp (°C)	C:Time (h)	Enzyme activity (Y) (IU / ml)
1	16	0.02	16	23	2.31
2	6	1	16	23	2.73
3	12	0.02	30	23	2.50
4	3	1	30	23	2.72
5	5	0.02	23	10	2.69
6	8	1	23	10	1.98
7	10	0.02	23	36	2.84
8	4	1	23	36	2.56
9	9	0.51	16	10	1.00
10	15	0.51	30	10	1.60
11	17	0.51	16	36	2.87
12	11	0.51	30	36	2.77
13	2	0.51	23	23	3.31
14	13	0.51	23	23	3.25
15	14	0.51	23	23	3.27
16	1	0.51	23	23	3.28
17	7	0.51	23	23	3.31

As a result, the coded variables response equation was obtained as following:

$$Enzyme\;activity\;(Y)=+3.28-0.044\times A+0.085\times B+0.47\times C-0.050\times AB+0.11\times AC-18\times BC-0.13\times A^{2}-0.59\times B^{2}-0.64\times C^{2}$$ (1)

Where 𝑌 is the actual response (FnCel5A activity in IU / mL), and 𝐴, 𝐵, and 𝐶 are the coded values for the independent variables (IPTG, induction temperature, and post-induction time respectively).

In addition, the actual variables equation was obtained as following:

$$Enzyme\;activity\;(Y)=-7.25619+0.41330\times IPTG+0.61604\times Temperature+0.24492\times Time-0.014577\times IPTG\times Temperature+0.016876\times IPTG\times Time-1.92308E-003\times Temperature\times Time-0.54456\times IPTG^{2}-0.012005\times Temperature^{2}-3.76183E-003\times Time^{2}$$ (2)

The ANOVA results in Table 4 confirm the accuracy of the model of FnCel5A activity. The quadratic type model was used, and the F-value 4.24 suggests the validity of the model. There is only a 3.50% possibility that an F-value larger could occur due to noise. Values of "Probability > F" less than 0.050 indicates that the model terms are significant. Here the significant model terms are C, B^**2**^ and C^**2**^ respectively. Values greater than 0.100 represent the model terms are insignificant. The lack of fit (F-value) of 496.04 justify that the lack of fit is significant. The predicted coefficient of determination (predicted 𝑅_2_ = ─1.474) is in reasonable correlation with the adjusted coefficient of determination (adjusted 𝑅_2_ = 0.6456). A negative predicted R squared suggests that the overall mean may be a best predictor of the response than the current model. Adequate precision measures the signal to noise ratio. A ratio greater than 4 is fit for the designed model. Here the ratio of 6.696 indicates an adequate signal. This model is applicable for the navigation of the design space.

**Table 4:** Estimated effect and ANOVA on Box-Behnken design for the three parameters.

Source	Sum of squares	Df	Mean square	F-value	p-value Prob > F	
Model	5.53	9	0.61	4.24	0.0350	significant
A-IPTG	0.015	1	0.015	0.11	0.7547	
B-Temp	0.058	1	0.058	0.40	0.5478	
C-Time	1.78	1	1.78	12.26	0.0100	
AB	1.000E-002	1	1.000E-002	0.069	0.8004	
AC	0.046	1	0.046	0.32	0.5899	
BC	0.12	1	0.12	0.85	0.3885	
A^2^	0.072	1	0.072	0.50	0.5038	
B^2^	1.46	1	1.46	10.05	0.0157	
C^2^	1.70	1	1.70	11.74	0.0110	
Residual	1.01	7	0.14			
Lack of Fit	1.01	3	0.34	496.04	< 0.0001	significant
Pure Error	2.720E-003	4	6.800E-004			
Cor Total	6.54	16				

Predicted R^2^ (─1.474), Adjusted R^2^ (0.6456), Df (degree of freedom), Adequate precision (6.696), significant (p ≤ 0.050), insignificant (p > 0.050.).

### Response surface analysis for influence of variables on *E*. *coli* culture

**Fig 1:**
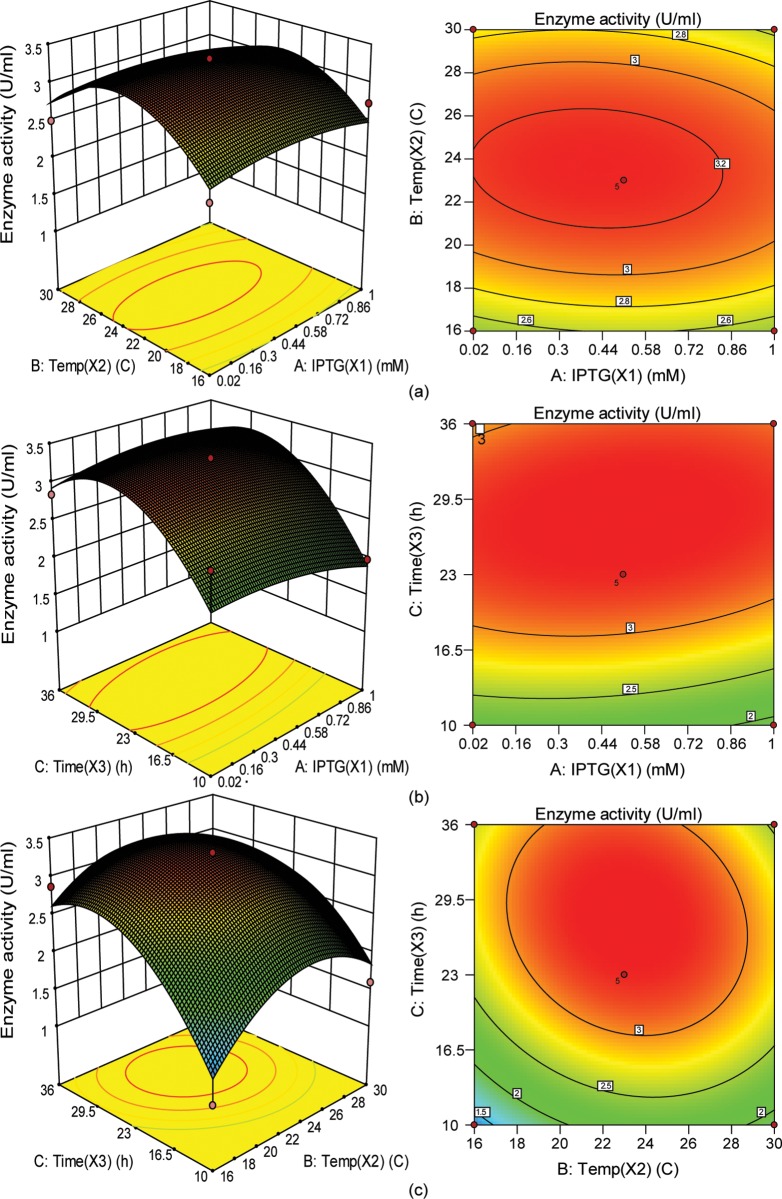
Response surface and respective contour plots. (a) Combined effect of IPTG and temperature (b) Combined effect of IPTG and time, and (c) Combined effect of temperature and time on FnCel5A activity.

To evaluate the effects of all the independent variables and their interactive effects on *E*.*coli* culture, three-dimensional response surface plots and the two-dimensional contour plots were drawn against two experimental variables while the other variable was kept constant as shown in Fig 1. Fig 1A shows the effect of modulating IPTG and temperature on FnCel5A activity while keeping time fixed. Increasing IPTG concentration from 0.02 mM to 0.86 mM and lowering the temperature from 30°C to 16°C significantly increased FnCel5A activity to 3.31 IU/mL. The three dimensional surface plot and its respective contour plot expedited the identification of the optimal values for IPTG and temperature, which were found to be 0.56 mM and 24°C respectively. Zhao *et al*. found that the maximum expression of interleukin 11 protein in *E*. *coli* was obtained at 0.5 mM IPTG [38], which is similar to our results for the production of endoglucanase FnCel5A. Here, we found that the optimal inducer concentrations for *E*. *coli* were far below the previously recommended inducer concentration of 1 mM IPTG [39]. This kind of inducible expression system allow the proper and adjustable segregation of the growth and production phases by the variation of the inducer concentration and the induction time [40].

Fig 1B showed the detail effects of IPTG and time, on FnCel5A activity. The results (Time, P = 0.0100, Table 4) showed that post-induction time has most significant effect as compared to IPTG (IPTG, P = 0.5478, Table 4). Wang *et al*. showed that induction time could influence the production of a newly recombinant sea anemone neurotoxin [41]. In the previous studies, the unoptimized induction time for this enzyme was a little shorter (20 hour) as compared to our optimized 29.5h induction time [10]. Fig 1C showing the interaction between time and temperature. These results showed that post-induction temperature is insignificant factor and has less effect on FnCel5A activity. Moradpour *et*, *al* also did not find any significant effect of the post induction temperature on the expression of recombinant cholesterol oxidase in *Escherichia coli* [42]. This effect can be observed from Fig 1C also that maximum activity of FnCel5A was obtained when the induction temperature was 24°C.

Our optimized model shows that maximum FnCel5A activity 3.31 IU/ml was obtained under the conditions with induction temperature of 24°C, IPTG concentration of 0.56 mM, and post-induction time of 29.5 hours. These actual values of optimization conditions were near the predicted values. These results showing that the model was appropriate for the production of FnCel5A.

### Perturbation plots for *E*.*coli* cell culture

**Fig 2:**
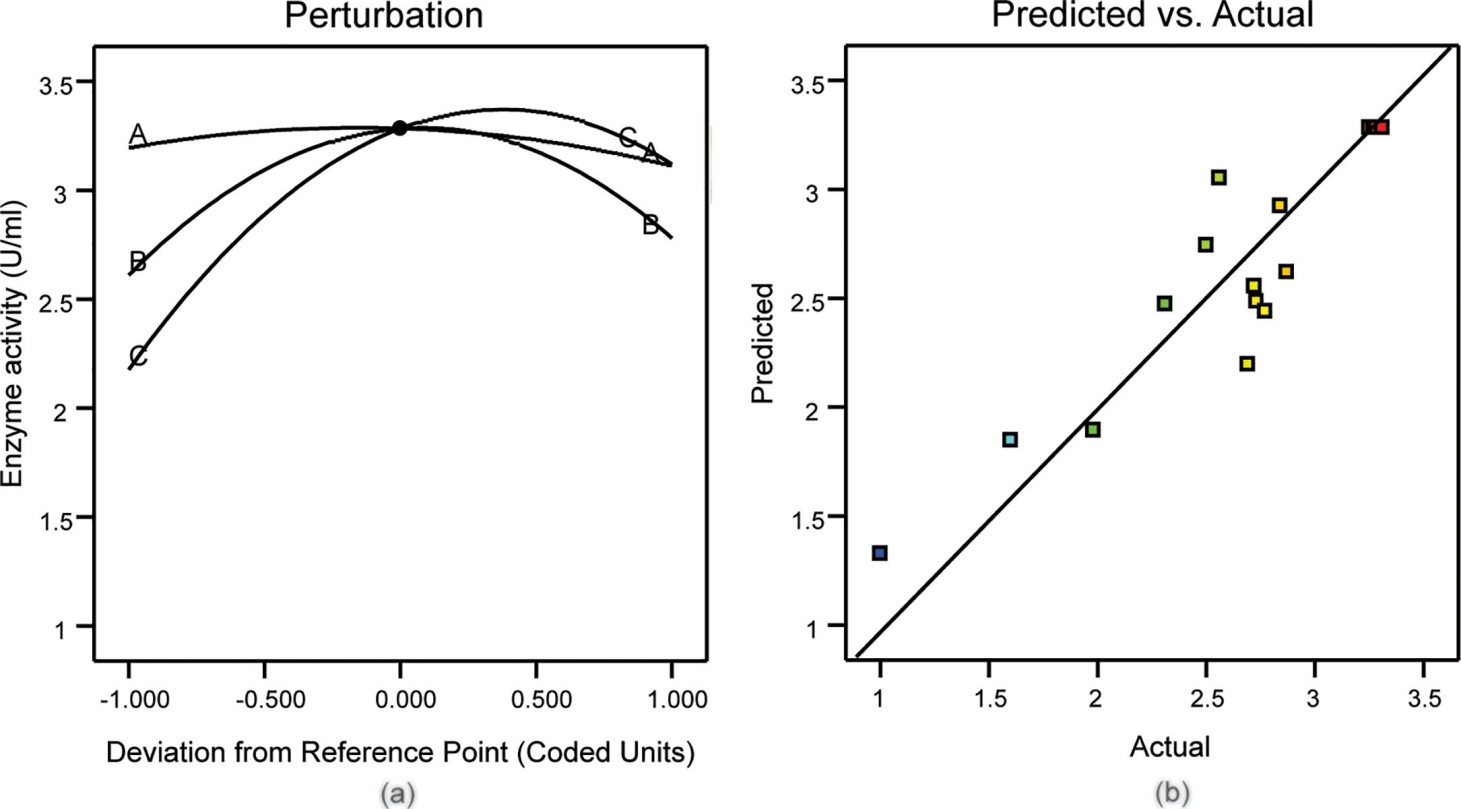
(a) Perturbation plot for *E*. *coli* culture (b) Predicted vs actual values of *E*. *coli* culture.

Perturbation plots illustrate the function of a specific factor when all the other parameters are fixed at their optimum levels [43]. The curvature or slope of the plot is indicative of the sensitivity of the tested factor [44]. In this study, perturbation plots Fig 2A were used to analyze the effect of each factor on *E*. *coli* culture. This analysis revealed that temperature and time influence the *E*. *coli* culture considerably by comparing the slope of each and every parameter, it became evident that time is the most dominant factor of the variables tested.

Comparing the predicted and actual plots Fig 2B for *E*. *coli* culture generated by the model equation showed tight clustering of the observed values along the straight line, which is a testament to their proximity to the predicted values (R^2^ = ─1.474). Taken together, these plots further prove the validity of the model for the prediction of *E*. *coli* culture dynamics.

### Box-Behnkan analysis of *E*.*coli* cell disruption

Intracellular recombinant proteins are traditionally produced by expression in bacterial host systems, particularly *E*. *coli*; therefore, maximizing protein recovery is crucial for industrial application [45]. The process of cellular disruption of *E*.*coli* was optimized based on a BBD using RSM for maximum recovery of recombinant, hyperthermophilic FnCel5A. The effect of pH, temperature and time on cell disruption and the resulting FnCel5A yield was demonstrated in this study. The combinations of these test variables and their resulting enzyme activities (IU/g) are presented in Table 5.

**Table 5:** Box-Behnken design arrangement for cell disruption and resulting activity.

Std	Run	Factor 1	Factor 2	Factor 3	Response (Y)
		A:Temperature (°C)	B:pH	C:Time (minutes)	Enzyme activity (IU/g)
1	4	70	6.21	17.5	9.14963
2	2	96	6.21	17.5	0.66664
3	14	70	8.21	17.5	12.4995
4	6	96	8.21	17.5	0.699972
5	8	70	7.21	5	10.5496
6	3	96	7.21	5	0.633308
7	16	70	7.21	30	11.8995
8	15	96	7.21	30	0.58331
9	17	83	6.21	5	8.63299
10	7	83	8.21	5	13.0495
11	10	83	6.21	30	94.3296
12	1	83	8.21	30	8.68299
13	11	83	7.21	17.5	13.9828
14	9	83	7.21	17.5	13.9328
15	12	83	7.21	17.5	13.9828
16	13	83	7.21	17.5	13.9661
17	5	83	7.21	17.5	13.9828

A regression equation yielded by statistical analysis depicting the empirical relationship between the test variables and their experimental response. As a result, the following quadratic equations for enzyme activity (Y) of coded and actual factors (Eqs 3 and 4 respectively) was obtained:

The coded variables response equation obtained as:

$$Enzyme\;activity\;(Y)=+13.97-5.19\times A+0.94\times B-0.34\times C-0.83\times AB-0.35\times AC-1.40\times BC-6.18\times A^{2}-2.04\times B^{2}-1.87\times C^{2}$$ (3)

While equation in terms of actual variables obtained as:

$$Enzyme\;activity\;(Y)=-376.03932+6.16869\times Temperature++37.54649\times pH+1.38043\times Time-0.063780\times Temperature\times pH-2.15369E-003\times Temperature\times Time-0.11224\times pH\times Time-0.036568\times Temperature^{2}-2.03548\times pH^{2}-0.011987\times Time^{2}$$ (4)

The results showed that the pH and temperature has significant effect on the *E*. *coli* cell wall breakage and protein release while the time has less effect.

ANOVA confirmed the accuracy of the model for optimizing *E*. *coli* cell lysis and FnCel5A as shown in Table 6. The F-value 92.67 suggests that the model is significant and only 0.01% chance may exist that an F-value larger could occur due to noise. Value of "Probability > F" less than 0.050 illustrate the significance of the model terms. In this model the terms, A, B, BC, A^2^, B^2^, and C^2^ are significant respectively, therefore, all these terms were included in the final model equation. The lack of fit (F-value) of 2615.90 suggests the lack of fit is significant. The predicted R-square of 0.8669 is in reasonable compliance with the adjusted R-square of 0.9810; i.e. the aberration is less than 0.2, which illustrates that 99.9% of the variability in the results can be elucidated by the model. Adequate precision measures the signal to noise ratio. According to the previous study a ratio larger than 4 is suitable. Our ratio of 24.957 showing a standard and fit signal. This analysis demonstrate the substantial coherence between the response and the significant independent variables as represented by the low model 𝑃 value (p < 0.05) and large lack-of-fit sum of squares value (441.82). The statistical significance of the six-modeled factors (A, B, BC, A^2^, B^2^, and C^2^) and the adequate precision as revealed by the model denotes a low signal to noise ratio. Among the linear terms, the most statistically significant effect on FnCel5A enzyme production resulted from pH and temperature.

**Table 6:** Estimated effect, contribution, and ANOVA of the three parameters.

	Sum of		Mean	(F	(p-value	
Source	squares	Df	square	value)	Prob > F)	
Model	441.82	9	49.09	92.67	< 0.0001	significant
A-Temperature	215.44	1	215.44	406.68	< 0.0001	
B-pH	7.02	1	7.02	13.26	0.0083	
C-Time	0.92	1	0.92	1.74	0.2292	
AB	2.75	1	2.75	5.19	0.0568	
AC	0.49	1	0.49	0.92	0.3682	
BC	7.87	1	7.87	14.86	0.0062	
A^2^	160.81	1	160.81	303.57	< 0.0001	
B^2^	17.45	1	17.45	32.93	0.0007	
C^2^	14.77	1	14.77	27.88	0.0011	
Residual	3.71	7	0.53			
Lack of Fit	3.71	3	1.24	2615.90	< 0.0001	significant
Pure error	1.889E-003	4	4.723E-004			
Cor total	445.52	16				

R^2^ (0.8669), Adjusted R^2^ (0.9810), Df (degree of freedom), sum of squares (441.82), significant (p ≤ 0.050), insignificant (p > 0.050.).

Numerous studies have proved that the membrane of bacteria can be damaged by heat treatment [46, 47]. Treatment at 55°C could partially disrupt the wild-type *E*. *coli*. When the temperature rises, the movement of lipopolysaccharide molecules in the outer membrane becomes increasingly drastic, this results in more efficient lysis [45]. With traditional methods, notably chemical treatment, the pH also contributes dramatically to both recombinant and total protein release from *E*.*coli* and reductions in pH result dramatically affect those outcomes [48]. Our results further establish that pH significantly influences protein release from *E*.*coli* cells, specifically the hyperthermophilic FnCel5A enzyme.

### Predicted vs. actual and normal plots of residuals for E.coli cell disruption

**Fig 3:**
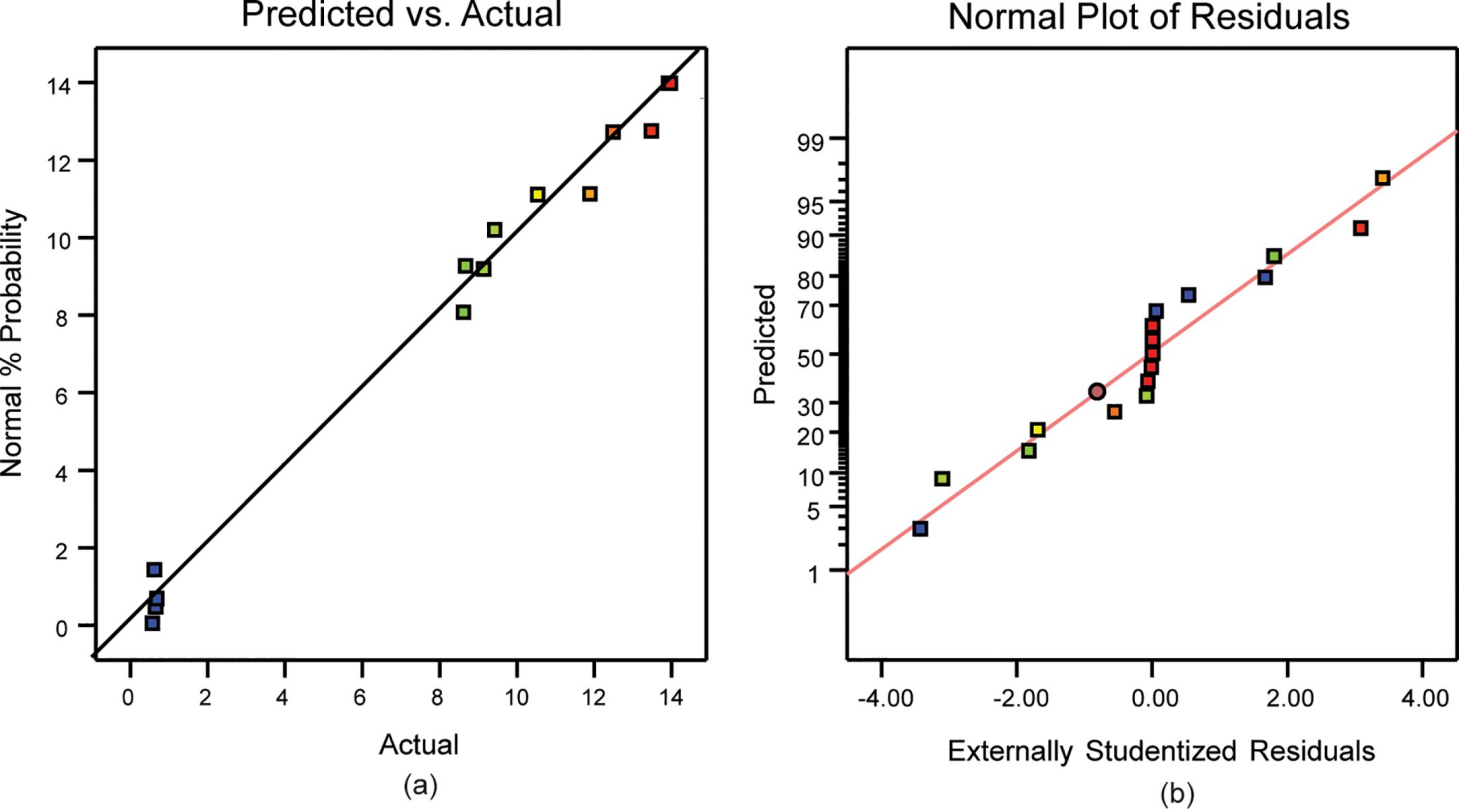
(a) Predicted vs. actual plot for for E.coli cell disruption. (b) Normal plot of residual for *E*.*coli* cell disruption.

Fig 3A, showing the actual versus predicted values and run order, respectively. The clustering of the runs near the straight line illustrates that there is very less difference in both the predicted and actual values. Similarly, Fig 3B demonstrates the close proximity between observed values and those predicted by the model, as illustrated by clustering near the straight line (R^2^ = 0.8669). In fact the coefficient of determination, R^2^ = 0.9057, considered as sufficient to determine the correlation between the predicted and actual values [49]. The values (both predicted and observed) for all the runs were close to each other. This agreement validates the model as suitable for prediction and optimization of enzyme production from *E*.*coli*.

### Analysis of response surface for influence of variables on E. coli cell disruption

**Fig 4:**
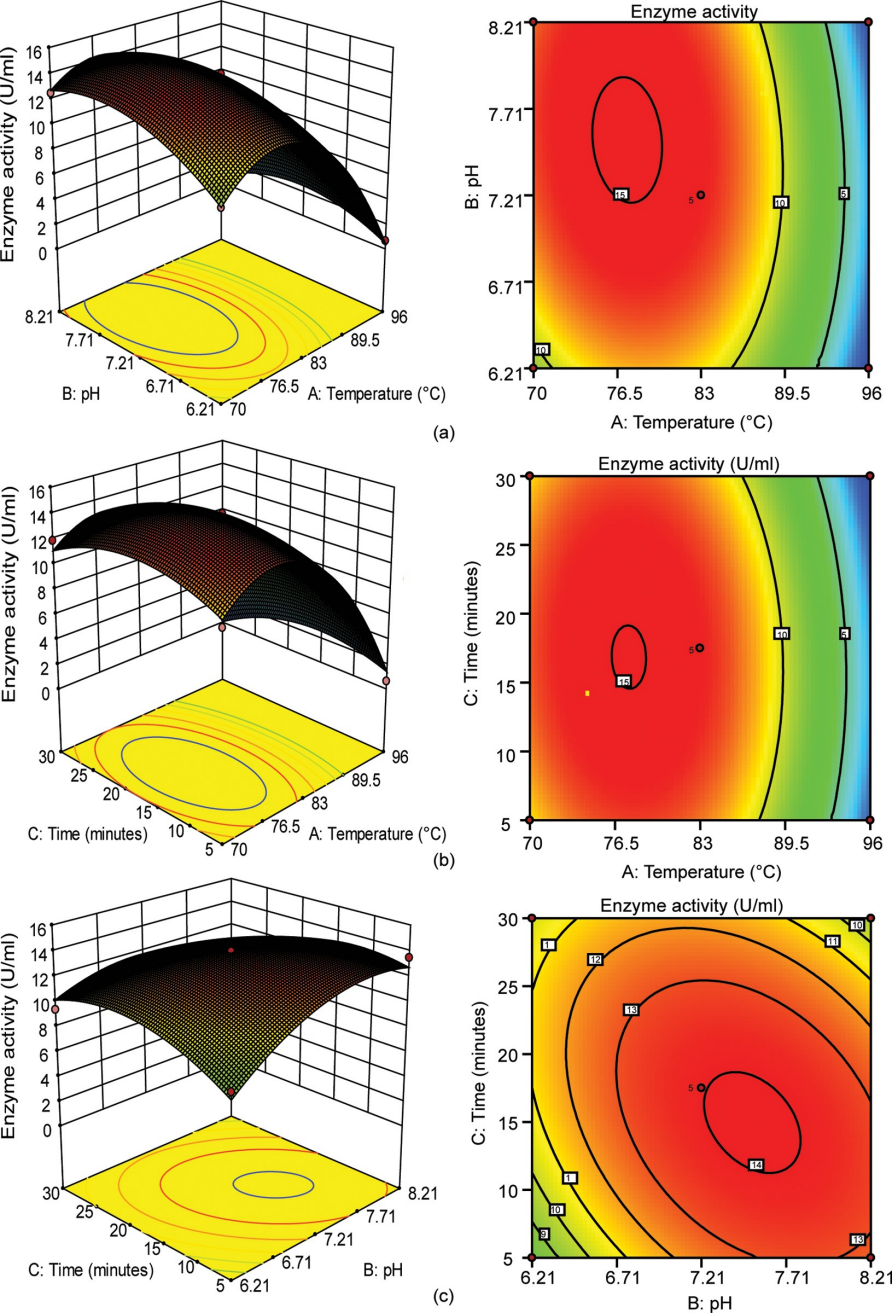
Response surface and respective contour plots for FnCel5A production. (a) The combined effect of temperature and pH (b) the combined effect of temperature and time, and (c) the combined effect of time and pH on FnCel5A production.

To examine the effects of the aforementioned three factors on FnCel5A yield, two-dimensional contour and three-dimensional surface plots were drawn. Fig 4 shows the contour and response surface plots for each pair of selected parameters while keeping the remaining factor constant at control levels. The impact of temperature and pH on FnCel5A production while time is held constant as shown in Fig 4A.The results show that the temperature and pH has the significant effect on the cell disruption of *E*.*coli*. The three dimensional and respective contour plots expedited the identification of optimal pH, incubation time, and temperature, which were 77°C, for 20 minutes at pH 7.71. Fig 4B and 4C illustrate the interaction of temperature and time, and pH and time on protein yield respectively, with the latter displaying the most statistically significant synergy. In the previously reported studies, the maximum esterase enzyme (75–91%) was released from 60 to 80 °C while the pH values ranging from 5 to 9 [48, 45]. In the previously reported methods of cell disruption, such as chemical treatment, the pH value often has dramatic effect on both recombinant and total protein release from *E*. *coli* cells. The change in pH involve can increase or decrease the recombinant and total protein release from the cells [48, 45]. All these studies confirmed the significance of each parameters and close relevance to the results of our optimized model.

### Experimental confirmation of E.coli culture and cell disruption

It is important to analyze the fitted model to ensure that it provides an appropriate estimation to the actual system. Unless the model is not showing an adequate fit, proceeding with the optimization of the fitted response surface will gave poor or ambiguous results [50]. The experiments confirmed that the suggested culture conditions were conducted according to the parameters suggested by the numerical model (induction temperature of 24°C, IPTG concentration of 0.56 mM, induction time of 29.5 hours). The effect of these factors was investigated towards validating the RSM study. As predicted, there was only a small difference between the predicted outcomes (3.20 IU/ml) and the observed enzymatic activity (3.31 IU/ml) when executed under the aforementioned optimal conditions.

In additional support of the model, the confirmatory experiments were conducted with the parameters suggested by the numerical modeling while keeping the temperature at 77°C, pH 7.71, and 20 minutes duration. This validation similarly showed only small differences between the predicted response (14.6 IU/g) and the observed activity (13.98 IU/g) under the aforementioned conditions. This confirmation illustrates the high degree of accuracy of the two optimal models, which is evidence of the validation of the model under the investigated conditions. Further, it confirms that the quadratic model is an adequate predictor of the experimental results. Finally, the combination of both optimized models generated 5772 IU/L yield, which is 74.3% higher than our previous single optimized culture model.

### Comparison of optimum thermolysis and bead milling process

Cell wall disruption is the cornerstone of the purification of intracellular proteins from *E*. *coli*. A comparative analysis of two different cell-disruption methods for the extraction of recombinant FnCel5A was therefore investigated: bead milling and the optimized thermolysis protocol. The yield from bead milling was 3310 IU/L less than half of the enzyme recovered using the optimized thermolysis protocol (5772 IU/L). According to these results, optimized thermolysis combined with optimized *E*. *coli* culture conditions were the most effective method for FnCel5A production. Moreover, the method optimized here is more straightforward, reproducible, cost effective, efficient, and less time consuming.

## Conclusion

A reliable and efficient process for the production of biofuels has gained momentum in recent decades. Development of thermostable cellulase has the potential to change the nature of the biofuel industry, but the efficient and low-cost production of thermostable cellulases is remain the major hindrance. Endo-β-1, 4-glucanase FnCel5A, a novel cellulase from glycosyl hydrolase family 5, is one of the most thermally resistant and catalytically efficient enzyme that exhibits the highest activity on carboxymethylcellulose among the reported counterparts, making it a promising candidate for development of industrial cellulose conversion processes. The main objective of this study was to reduce the production cost of this enzyme. By using statistical methodologies, we optimized the fermentation and cell-disruption protocol that resulted in the production of 5772 IU cellulase per liter LB culture, which is the highest yield of this enzyme as far as we know. In summary, our optimization platform explored in this study provide a more dynamic and more economical production process for the industrial-scale applications of thermostable celluase.
